# THE USE OF SOLUTION-ORIENTED STRATEGIES TO SUPPORT GRANDPARENTS RAISING GRANDCHILDREN

**DOI:** 10.1093/geroni/igz038.1294

**Published:** 2019-11-08

**Authors:** Julian Montoro-Rodriguez, Bert Hayslip, Jennifer Ramsey, Jane l Jooste

**Affiliations:** 1 University of North Carolina at Charlotte, Charlotte, North Carolina, United States; 2 University of North Texas, Denton, Texas, United States; 3 University of North Carolina - Charlotte, Charlotte, North Carolina, United States; 4 Lewisville ISD Special Education, Purnell Support Center, Lewisville, Texas, United States

## Abstract

There is consistent evidence that caregiving for grandchildren is psychologically, emotionally and physically challenging for grandparents. However, many of them may not be able to receive the support they need from their own families or public services. Using the Selective Optimization with Compensation framework and principles of solution-oriented cognitive models, we implemented a six-week session pilot program to increase grandparents’ effective coping strategies, reduce stress, and improve parenting and communication with grandchildren. Preliminary data from 22 grandparents suggest that participants improved their use of effective selection strategies to set priorities and pursue goals related to their wellbeing and the quality of their relationship with grandchildren. Their pre-post test scores also indicated a higher level of parental efficacy, social relations and confidence in organizing their time and priorities. Results indicate that focusing on solution-oriented strategies may empower grandparents to improve their psychological adjustment and take care of their needs.

